# Changing Flannel

**Published:** 1876-02

**Authors:** 


CHANGING FLANNEL.—Many persons fall into serious disease by changing their winter flannels too soon. The first of May is quite early enough to lay them aside for a thinner material, south of Virginia, Tennessee, and Kentucky; but the people of those States, and north of them, should not make the change earlier than the last of May. In New York, it is sometimes cold enough for a fire on the first of June. Silk woollen, or cotton flannel should be worn next the person of all throughout the year.

